# Maternal and Live-birth Outcomes of Pregnancies following Assisted Reproductive Technology: A Retrospective Cohort Study

**DOI:** 10.1038/srep35141

**Published:** 2016-10-20

**Authors:** Linling Zhu, Yu Zhang, Yifeng Liu, Runjv Zhang, Yiqing Wu, Yun Huang, Feng Liu, Meigen Li, Saijun Sun, Lanfeng Xing, Yimin Zhu, Yiyi Chen, Li Xu, Liangbi Zhou, Hefeng Huang, Dan Zhang

**Affiliations:** 1 Department of Reproductive Endocrinology, Women’s Hospital, School of Medicine, Zhejiang University, Hangzhou, Zhejiang, 310006, P. R. China; 2 Key Laboratory of Reproductive Genetics (Ministry of Education), Zhejiang University, Hangzhou, 310006, P. R. China

## Abstract

This study was carried out to explore associations between assisted reproductive technology (ART) and maternal and neonatal outcomes compared with similar outcomes following spontaneously conceived births. We conducted a retrospective cohort study of pregnancies conceived by ART (N = 2641) during 2006–2014 compared to naturally conceived pregnancies (N = 5282) after matching for maternal age and birth year. Pregnancy complications, perinatal complications and neonatal outcomes of enrolled subjects were investigated and analysed by multivariate logistic regression. We found that pregnancies conceived by *in vitro* fertilization (IVF) were associated with a significantly increased incidence of gestational diabetes mellitus, gestational hypertension, preeclampsia, intrahepatic cholestasis of pregnancy, placenta previa, placental abruption, preterm premature rupture of membranes, placental adherence, postpartum haemorrhage, polyhydramnios, preterm labour, low birth weight, and small-for-date infant compared with spontaneously conceived births. Pregnancies conceived by intracytoplasmic sperm injection (ICSI) showed similar elevated complications, except some of the difference narrowed or disappeared. Singleton pregnancies or nulliparous pregnancies following ART still exhibited increased maternal and neonatal complications. Therefore, we conclude that pregnancies conceived following ART are at increased risks of antenatal complications, perinatal complications and poor neonatal outcomes, which may result from not only a higher incidence of multiple pregnancy, but also the manipulation involved in ART processes.

As a result of advances in technology and provision of services, an increasing number of infants are born as a result of assisted reproductive technology (ART) therapy. In developed countries, ART pregnancies represent 1.7% to 4.0% of all births^1^, while 1.0% of all births in China are the result of ART^2^.

A “good perinatal outcome” among live births after ART is defined as the live birth of a singleton infant born at term (≥37 completed weeks of gestation) and at a normal birth weight (≥2,500 g)^3^. However, concern is mounting over the safety of ART and its effect on maternal and fetal well-being. It is well documented that ART pregnancies have a significantly higher risk of multiple pregnancy and adverse perinatal outcomes, including preterm delivery, low birth weight, and birth defects^4^,^5^,^6^. Some studies have suggested an increased risk of preeclampsia, gestational hypertension, placenta previa, and gestational diabetes in ART pregnancies^2^,^7^,^8^.

Nevertheless, there is scarce data examining the type of ART used in relation to the maternal and live-birth outcomes. Also, many prior studies failed to control for maternal age and other relevant variables, for example, gravidity and parity history. The present retrospective cohort study was undertaken to compare the risks of pregnancy complications, perinatal complications and neonatal outcomes in pregnancies following different types of ART with matched spontaneously conceived pregnancies, and in addition to explore the relationship between ART and adverse outcomes with discussion of the underlying mechanisms.

## Results

### Maternal Characteristics in Pregnancies Conceived after ART and Spontaneity

From 2006 to 2014, 2641 ART-derived pregnancies and 5282 matched spontaneous pregnancies were enrolled in our study. The ART group was divided into *in vitro* fertilization (IVF) subgroup (n = 2327) and intracytoplasmic sperm injection (ICSI) subgroup (n = 314). The ART group consisted of 1659 singleton gestations and 982 twin gestations, while the spontaneously conceived group consisted of 5193 singleton gestations and 89 twin gestations. Table 1 summarized the maternal and prenatal characteristics. The IVF/ICSI patients in this study tended to be nulliparous, and had higher body mass indices (BMI) and yet a lower rate of previous cesarean delivery. ART births were closer to 20 times more likely to be twins. 2117 women (91%) who conceived after IVF and 209 pregnant women (66.6%) by ICSI had their babies by cesarean section.

### Pregnancy, Perinatal Complications and Neonatal Outcomes in ART and Spontaneously Pregnant Groups with Stratified Analysis by Birth Plurality

It was demonstrated that women undergoing ART were more likely to develop pregnancy complications during pregnancy (Table 2). Pregnancies after ART were 1.99 times more likely to develop gestational diabetes mellitus (GDM) (95% CI 1.69–2.36), 2.58 times more likely to have gestational hypertension (95% CI 2.11–3.15), 1.49 times more likely to develop preeclampsia (95% CI 1.12–1.98), and 2.86 times more likely to develop intrahepatic cholestasis of pregnancy (ICP) (95% CI 2.39–3.42) compared with controls. In singleton gestations, the incidence of GDM, gestational hypertension and ICP was still significantly higher than those of the controls. However, there was no statistically significant difference in the incidence of these obstetric complications in twins.

An association between ART and perinatal complications was also significant, which should not be neglected (Table 2). Nearly all the perinatal complications listed, including placenta previa (OR, 2.23, 95% CI 1.79–2.78), placental abruption (OR, 5.06, 95% CI 2.83–9.06), preterm premature rupture of membranes (pPROM) (OR, 3.05, 95% CI 2.48–3.74), placental adherence (OR, 2.37, 95% CI 1.90–2.95), postpartum haemorrhage (OR, 2.72, 95% CI 2.18–3.41), and polyhydramnios (OR, 1.79, 95% CI 1.26–2.53), were more likely to occur after ART in all birth, as well as in singletons with the exceptions of preeclampsia, abnormal placental cord insertion, and oligohydramnios. Nevertheless, no significantly increased incidence of perinatal complications, apart from postpartum haemorrhage, was observed in twins on comparison of the two groups.

The proportions of preterm labour, low birth weight, small for date infant, 1-minute Apgar ≤ 7 and 5-minute Apgar ≤ 7 were significantly higher for ART births than spontaneously conceived births in the total births (Table 3). The incidence rate of fetal macrosomia in the ART group was 3.3%, significantly lower than that in the control group. The differences in macrosomia, small for date infant, and 1-minute Apgar ≤ 7 were not statistically significant after restriction to singletons.

### Pregnancy, Perinatal Complications and Neonatal Outcomes in IVF, ICSI and Spontaneously Pregnant Groups

We futher studied the incidences of pregnancy and perinatal complications related with different type of ART, including IVF and ICSI (Table 4). As expected, patients who underwent IVF were more likely to suffer from GDM (11.7%), gestational hypertension (9.3%), preeclampsia (4.0%), ICP (11.8%), placenta previa (7.1%), placental abruption (1.6%), pPROM (9.9%), placental adherence (7.7%), postpartum haemorrhage (7.3%), and polyhydramnios (2.2%), compared with spontaneously conceived pregnancies. We also observed a decrease in the incidence of oligohydramnios in pregnancies undergoing IVF. ICSI pregnancies exhibited higher rates of GDM, gestational hypertension, ICP, pPROM, and postpartum haemorrhage in comparison with spontaneous pregnancies. It seemed that ICSI did not bring more extra pregnancy complications than IVF.

The neonatal outcomes of the three groups were summarized in Table 5. Statistically significant increases were noted in preterm labour (39.9%), low birth weight (37%), and small for date infant (3.6%) among infants who were born after IVF treatment, as well as 1-minute Apgar ≤ 7(5.1%) and 5-minute Apgar ≤ 7 (0.9%). Also, the ICSI group had more negative neonatal outcomes (preterm labour, low birth weight, and small for date infant) than the control group. A decreased incidence of infants with macrosomia was found in the IVF and ICSI group. Differences became insignificant when comparing the incidence between IVF subgroup and ICSI subgroup, apart from preterm labour.

### Pregnancy, Perinatal Complications and Neonatal Outcomes in Nulliparous and Multiparous Groups

The incidences of pregnancy and perinatal complications in nulliparous and multiparous women were exhibited in Table 6. There were 2392 ART nulliparous women and 4328 spontaneously pregnant nulliparous women in our study. In the ART nulliparous group, 260 women had a pregnancy complicated by GDM (10.9%), compared with 269 women in the spontaneously pregnant nulliparous group (6.2%, P < 0.001). Significant rises of incidence were also observed in gestational hypertension, preeclampsia, ICP, placenta previa, placental abruption, pPROM, abnormal placental cord insertion, placental adherence, postpartum haemorrhage and polyhydramnios in ART nulliparous women. Meanwhile, among the 249 ART multiparous women and 954 spontaneously pregnant multiparous women, the risk of GDM, gestational hypertension, ICP, placental adherence, and postpartum haemorrhage in ART multiparous women were also markedly increased.

The incidences of poor newborn outcomes in ART nulliparous and multiparous women were shown in Table 7. Statistically significant increases were noted in preterm labour (39.4%), low birth weight (36.8%), small for date infant (4.0%), 1-minute Apgar ≤ 7(4.9%) and 5-minute Apgar ≤ 7 (1.3%) in ART nulliparous women, compared to spontaneously pregnant nulliparous women. On the contrary, there was a decline in the incidence of macrosomia in ART nulliparous group. The occurring rates of preterm labour (42.2%) and low birth weight (33.4%) were significantly elevated in ART multiparous group, compared to spontaneously pregnant multiparous women, with no significant difference in small for date infant, 1 minute Apgar  ≤ 7 and 5 minute Apgar ≤ 7.

We then compared the incidences of pregnancy, perinatal complications and infant outcomes in singletons of nulliparous group conceived with ART and conceived spontaneously (Table 8). Totally, there were 1493 ART nulliparous singletons and 4255 spontaneously pregnant nulliparous singletons. In the ART nulliparous singletons group, 177 women had a pregnancy complicated by GDM (11.9%), compared with 262 women in the spontaneously pregnant nulliparous singletons (6.2%), suggesting that ART nulliparous singletons had a greater risk of GDM (P < 0.001). Similarly, statistically significant elevated risks of pregnancy complications including gestational hypertension, preeclampsia, ICP, placenta previa, placental abruption, pPROM, Abnormal placental cord insertion, placental adherence, postpartum haemorrhage, and polyhydramnios were observed in ART nulliparous singletons. However, no statistically significant difference was shown in the incidence of oligohydramnios.

ART nulliparous singletons exhibited significantly increased rates of preterm labour (17.1%), low birth weight (10.3%), 1-minute Apgar ≤ 7 (3.8%) and 5-minute Apgar ≤ 7 (0.7%). compared with spontaneously pregnant nulliparous singletons. Nevertheless, the differences in small for date infant and macrosomia were not statistically significant between the two groups.

## Discussion

Despite the wide spread application of ART, studies focused on the obstetric, perinatal, and neonatal outcomes following ART are limited, and the previous studies remain controversial, partly because of different study designs, populations and countries. The present retrospective, hospital-based cohort study containing 7923 subjects was carried out from 2006 to 2014, and corroborated the increased risks of pregnancy complications, perinatal complications and neonatal poor outcomes related to ART. A 20-fold increased incidence of twin gestations in ART pregnancies compared with spontaneous pregnancies was found, and the stratified analysis by birth plurality was then carried out. Furthermore, associations between different types of ART, gravidity and parity history and adverse maternal and live-birth outcomes were investigated.

After restriction to singletons, these rates were reduced, consistent with previous researches^9^,^10^, suggesting the necessity to limit the number of embryo transferred legally and to promote single embryo transfer (SET) by the national policy guide. In the ART singleton gestations, 124 women had a pregnancy complicated by gestational hypertension (7.5%), as compared with 125 women in the ART twin gestations (12.7%), suggesting that ART multiples were associated with an increased risk of gestational hypertension. However, singletons of ART pregnancy still exhibited increased maternal and neonatal complications as well. These findings highlight the importance of better IVF process management, and provide valuable information for estimating and improving the safety of ART.

In the present study, we found that an increased risk of hypertensive disorders in pregnancy was associated with pregnancies conceived by ART: gestational hypertension (2.58-fold), preeclampsia (1.49-fold). To date, a wealth of studies had reported that women conceived by ART were at an increased risk for preeclampsia^7^,^11^,^12^, which was consistent with our study. The associations were reduced when analyses were limited to singletons, while significant differences yet remained. Preeclampsia and eclampsia, the etiology of which remains unclear, are major causes of maternal and perinatal mortality and morbidity. Recent studies suggested an association between preeclampsia and defective placental vascular remodeling, abnormal genetic polymorphisms, immune intolerance, vascular endothelial cell activation, and exaggeration of a systemic inflammatory process^13^,^14^. The mechanisms by which ART might lead to the increased risk for preeclampsia were elusive. While Chen XK *et al.*^12^ reported that there was no increased incidence of preeclampsia among pregnancies conceived by controlled ovarian hyperstimulation (COH) and intrauterine insemination, ART procedure itself might contribute to the marked increased incidence of preeclampsia. Future researches to further delineate placental development in ART births are needed to reveal the underlying mechanism.

GDM results from abnormal glucose tolerance and insulin resistance during pregnancy. The incidence of GDM in our ART group was 11.7%, 1.99-fold increase compared with controls. After restricting to singletons, the incidence increased slightly to 12.9%. Reddy *et al.*^15^ and Allen *et al.*^16^ also reported that pregnancies after ART demonstrated increased rate of GDM. It is clear that ART is an independent risk factor for GDM. The etiology of GDM is not yet clear. It is suggested that insulin resistance and hyperinsulinemia might partly explain the etiology of GDM^17^. GDM could be related to the relatively high prevalence of polycystic ovary syndrome (PCOS) among patients undergoing ART^18^. Furthermore, insulin resistance, which is a known risk factor for the development of GDM, has been proven in a certain proportion of PCOS patients. Moreover, several human studies found that active demethylation might be induced by ART^19^,^20^ while epigenetic changes were likely to be involved in GDM. The development of GDM is proposed to be resulted from epigenetic modifications. Well-designed multiple-center longitudinal and intervention-based studies would be helpful and are need in the future.

Our study also found that pregnancies with ART had a higher rate of ICP (11.4% vs. 4.3%, for with ART and no ART, respectively). We compared the risk of ICP in singletons and twins between the ART and spontaneous groups, and the results showed increased risk only in singletons (OR, 1.59, 95% CI 1.25–2.02). Given the low incidence of ICP for most areas, there were few earlier researches on associations between ART and ICP^21^. The disease appears to be more prevalent among pregnant women in China and it has been associated with increased rates of fetal morbidity and mortality, and an increased risk of maternal coagulopathy. The national conditions underscore the need for study of the possible link between ART and ICP.

We had established that women who underwent ART were more likely to have placenta-mediated pregnancy complications, which comprised a group of diseases, including placenta previa, placenta abruption, abnormal insertion of umbilical cord, and placental adherence. Compared with spontaneous conceptions, ART singleton pregnancies were associated with significantly higher odds of placenta previa (OR, 2.25, 95% CI 1.75–2.89), placenta abruption (OR, 4.43, 95% CI 2.28–8.61), and placental adherence (OR, 2.21, 95% CI 1.71–2.84). This interesting finding raises the possibility that intrauterine operation and manipulation of embryonic cells in ART might be related to abnormalities of location, development and function of the placenta. Shevell *et al.*^11^ observed an increased incidence of abnormal placentation in IVF pregnancies, and supposed that initiating of pregnancy and chorion formation *in vitro* might be the reasons for these complications during gestation. Romundstad *et al.*^22^ compared the risk of placenta previa between consecutive pregnancies in the same mother, where one sibling was conceived spontaneously and the other by IVF, and found that placenta previa occurred six times more often in singleton pregnancies after assisted reproduction. Only a few studies have been conducted to evaluate the possible differences in abnormal placental cord insertion and placental adherence among women who delivered after ART with matched controls of spontaneous pregnancies. In the present study, we found that placental adherence and abnormal placental cord insertion occurred more frequently in the ART group. Placental adherence reflects abnormal development of the placenta, and it is an independent risk factor for postpartum haemorrhage, affecting maternal and fetal health. Abnormal placental cord insertion, classified as marginal, and velamentous, may induce intrauterine growth restriction in IVF twins^23^. Still, it is reasonable to assume that the abnormal placental cord insertion plays a role in adverse neonatal outcomes. Our group has been considering future study to further explore the underlying mechanism. Earlier studies implied that high incidence of placental features in the IVF group may be related to inadequate orientation and/or superficial implantation of the blastocyst due to intrauterine embryo transfer^24^, and trophectodermal cells might be more sensitive to preimplantation epigenetic upset than inner cell mass^25^. Taking those studies into consideration, it was suggested that abnormal material exchanges at maternal-fetal interface resulting from inadequate or abnormal placental development might be the reason for poor maternal and live-birth outcome events.

Additionally, it was clear from the study that ART carried an increased risk of polyhydramnios. Findings from our study also indicated a decreased risk of oligohydramnios, not meeting agreements with previous study^26^. The investigation of this is seldom and the underlying mechanisms were uncertain. One possible explanation was regulating disorder of amniotic fluid production, transportation and absorption, leading to the unbalance of the amniotic fluid volume. The mechanisms leading to these adverse outcomes require a multicenter study and fundamental research for elucidation.

Besides, there were significantly more ART pregnant women diagnosed with pPROM compared to the controls (9.8% vs. 3.3% in total births, 5.1% vs. 3.1% in singletons, respectively). Although its causes remained unknown, pPROM after ART, is the crucial factor for preterm labour and low birthweight. This finding should arouse more recognition of the link between ART and pPROM.

In accordance with other authors, higher rates of preterm birth, low birthweight, and small for dates infant were observed in the ART group. 38.8% gestations conceived following ART were born preterm, 36.5% infants were low birthweight, and 3.8% were small for dates. This phenomenon was thought to result from multiple births in the view of clinicians and researcher. It has previously been suggested that twin pregnancies represents the key factor in the adverse complication for both mother and newborns after IVF/ICSI treatment^27^. Our observations support these results. The prevalence of twin pregnancies was as high as 37.7% in IVF and 33.4% in ICSI. The risk of adverse outcomes was much higher for both pregnant women and children born from multiple pregnancies. Currently, many clinicians remain to be convinced that single embryo transfer is a better clinical option to lower multiple pregnancy rate, with its following adverse outcomes^28^,^29^. Interestingly, an analysis in Australian and New Zealand assisted reproduction programs showed that liveborn singletons conceived by double embryo transfer (DET) had significantly lower mean birthweight and a higher rate of preterm birth than singletons conceived by SET^30^, which supported the suggestion that increase in proportion of SET procedures would result in a lower rate of multiple births and better perinatal outcomes, even in singletons. Although the incidence of multiplets following ART in China is significantly higher than that of North America and European countries^31^,^32^, we surely believe that, with the advancement of ART technology and the strengthen of national guidance, SET will be the top choice in the days to come.

Romundstad *et al.*^33^ reported that in the sibling-relationship comparisons, birthweight did not differ substantially between women who conceived spontaneously or after ART, and might therefore be caused by the underlying infertility, rather than ART procedures. Nevertheless, some other researchers held the view that ART procedures might be a major cause of the increased adverse maternal and live-birth outcomes^34^. There are still a lot of unsolved problems with regard to ART, and we need to make its molecular and cellular mechanism clear. Our study showed an increased risk for preterm labour and low birthweight in both total ART group and singleton group compared with the corresponding controls, but unexpectedly, we did not observe an increase in the incidence of preterm labour in twin pregnancies. The reasons for that were probably: (1) lack of enough samples of twin pregnancies with spontaneously achieved pregnancies; (2) most of the twins conceived by DET are dizygotic, while most spontaneously conceived twins are monozygotic. Therefore, we speculated that it was the result of statistical deflection instead of the advantage of assisted fertilization.

In the IVF subgroup, rates of most pregnancy-induced diseases, perinatal complications and infant outcomes were increased. However, when it came to ICSI, in many instances, the difference narrowed or even disappeared. This finding might be explained by the lower rate of twin gestation in ICSI or limited sample size of women undergoing ICSI. Zollner *et al.*^35^ found an increased risk of high blood pressure, preeclampsia, growth retardations and bleeding after IVF pregnancies as well as premature births and intrauterine deaths, attributing more to multiples and to the risk factors of the women involved, rather than the technology itself.

Due to the previous one-child policy in China, only a small number of multipara cases were included in this study. Although complications of ART in multiparas had a similar increased trend in the comparison of nulliparous, the difference of many complications were not statistically significant. With the releasing and carrying out of two-child policy in 2016, the number of multiparas undergoing ART is expected to increase in the coming years. We will continue to follow up more multiparas to enlarge our sample size.

Since the above logistic regression revealed that parity might influence pregnancy outcome, and multiple gestations have negative effects on pregnancy outcomes, we eliminated the cases of twins and multiparas to compare ART nulliparous singletons and spontaneously pregnant nulliparous singletons. We found that ART nulliparous singletons still had higher risks of pregnancy and perinatal complications, compared with spontaneously pregnant nulliparous singletons, implying that some potential factors, such as *in vitro* ART process or maternal factors might impact the pregnant outcomes. A meta-analysis demonstrated that ART singleton pregnancies were associated with higher risks of adverse obstetric outcomes which need obstetricians spend more concerns on perinatal stage in ART singleton pregnancy^26^. Our results that ART nulliparous singletons had increased rates of preterm labour, low birth weight and 5-minute Apgar scoring confirmed the above-mentioned statements. Previous researches showed maternal characteristics of subfertile women were associated with a lower birthweight, rather than *in vitro* fertilization treatment itself additionally contributed to a lower birthweight in the offspring^36^. However, there was no difference in the incidence of macrosomia and small-for-date infant between the two groups in our study, which might be the result of good nutritional status.

Limitations in our study cannot be overlooked, since no data were collected on the particular cause of infertility, baseline endocrine level, ovarian stimulation protocol, serum hormone levels during ovarian stimulation, as well as number and quality of embryos transferred. Therefore we cannot comment on how these different factors may affect maternal and neonatal outcomes. Ovarian stimulation might be associated with a greater incidence of adverse outcomes than natural cycles, and ovarian stimulation protocol and the hormone levels during ovarian stimulation might be an important confounding variable. The intrauterine insemination (IUI) is a relatively less invasive form of ART. The analysis of the maternal and neonatal outcomes of IUI with ovarian simulation versus natural cycles should be helpful for testing the theoretical paradigm that the more intricate and invasive the form of ART used, the more likely is the pregnancy outcome to be adverse. However, the records of IUI outcomes are lacked in our database, and not included in the present study. It should be considered in future research plan.

In summary, ART births are strongly associated with poorer maternal and live-birth outcomes. Multiple pregnancies can partly explain this phenomenon. However, ART nulliparous singletons still exhibited higher risks of pregnancy and perinatal complications, compared with spontaneously pregnant nulliparous singletons. Elective single embryo transfer should be strongly advocated to reduce the obstetrical risks of ART pregnancy. Since singletons born after the use of ART do worse than those conceived spontaneously, it is suggested that ART process itself is also significantly related to pathologic pregnancy, especially abnormal placental development. Given our findings, we suggest the following: (1) strict control of indications for ART (2) promoting SET; (3) improve the safety of manipulation in the ART process; (4) strengthen antenatal care of ART pregnancies. Whether these adverse outcomes are attributed to couples’ subfertility or ART itself need to be investigated further.

## Materials and Methods

### Study Population

**Figure 1:**
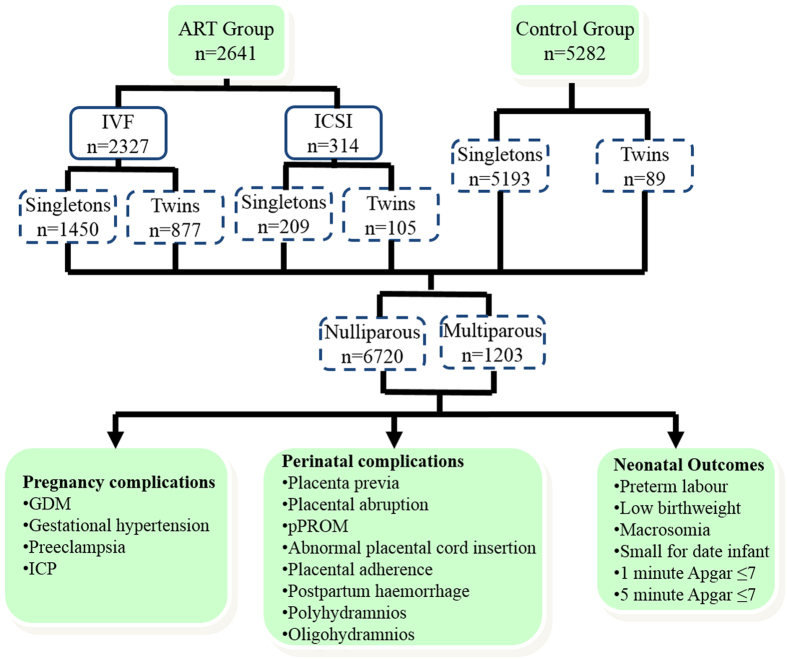
Flow chart of context diagram in the study. (**a**) IVF vs. ICSI vs. Control Group. (**b**) ART Total Group vs. Control Total Group; ART Singletons vs. Control Singletons; ART Multiples vs. Control Multiples. (**c**) ART Nulliparas vs. Control Nulliparas; ART Multiparas vs. Control Multiparas. (**d**) ART Nulliparous Singletons vs. Control Nulliparous Singletons.

This study is a retrospective, hospital-based cohort study, carried out at Women’s Hospital, School of Medicine, Zhejiang University between January 2006 and December 2014. All 2641 ART-derived pregnancies were matched in a 1:2 fashion to a random sample of spontaneous pregnancies for maternal age and birth year. ART group consisted of 2327 cases of IVF and 314 cases of ICSI. The mean (±SD) maternal age was 31.87 ± 3.96 years in the IVF group, 31.62 ± 3.98 years in ICSI group and 31.72 ± 3.21 years in the control group (Table 1). The ART group and control group were compared for the rates of pregnancy, perinatal complications and neonatal outcomes. Then we further conducted the stratification analysis by types of ART, birth plurality and parity. Figure 1 presents the study flow chart.

Available information in this dataset included maternal and prenatal factors (maternal age, gravidity with a range of 1 to 11, parity with a range of 1 to 6, birth plurality, maternal education, health problems, previous cesarean delivery, caesarean section, pre-pregnant BMI), and birth outcome (gestational age, birth weight, Apgar score). Clinical definition of the related complications and outcomes were listed in Supplementary Table S1. All data were retrospectively collected on a computerized database or by telephone interview. This retrospectively was approved by the Institutional Review Board of Women’s Hospital, School of Medicine, Zhejiang University. Informed consent was obtained from all patients.

Only data from live newborns after the 28^th^ week of gestation were included in the analysis. Donor oocytes/sperm or embryo recipients, ovulation induction or women applied preimplantation genetic diagnosis were excluded. All subjects with chronic hypertension (hypertension that predated or was diagnosed before the 20^th^ week of gestation), diabetes (insulin dependent or noninsulin dependent diabetes occurred before gestation), or heart disease (any preexisting cardiac diseases including dysrythmias, congenital anomalies, etc.), or fetal anomalies were excluded from this study, because they might be important confounding variables in the observed associations. The methods were carried out in accordance with the approved guidelines.

### Statistical analysis

Fisher’s exact probability test was used to compare categorical data. F test was used to evaluate statistical significances of continuous parametric data. Adjusted odds ratios (OR) with 95% confidence intervals (CI) were calculated to approximate relative risks of adverse outcomes. Odds ratios, adjusted for gravidity (1, ≥2), parity(1, ≥2), maternal education (<secondary school, secondary school, collage grade, post-graduate), previous caesarean section (yes, no) and BMI (<18.5, 18.5~23.9, 24.0~27.9, ≥28), were estimated using multivariate logistic regression. The method of backward LR was used for the selection of independent variables in logistic regressions with entry p-value = 0.05 and removal p-value = 0.1. The equations of logistic regression were described in Supplementary Table S2. P values of less than 0.05 were considered statistically significant. Bonferroni correction was employed in our data analyses in multiple comparisons. SPSS software (Version 16, Chicago, IL, USA) was used for data analyses.

## Additional Information

**How to cite this article**: Zhu, L. *et al.* Maternal and Live-birth Outcomes of Pregnancies following Assisted Reproductive Technology: A Retrospective Cohort Study. *Sci. Rep.* **6**, 35141; doi: 10.1038/srep35141 (2016).

## Supplementary Material

Supplementary Information

## Figures and Tables

**Table 1:** Maternal Characteristics among ART and Spontaneously Pregnant Groups.

	ART (n = 2641)		Controls (n = 5282)
	IVF (n = 2327)	ICSI (n = 314)	
Maternal age (years)	31.87 ± 3.96	31.62 ± 3.98	31.73 ± 3.21
Gravidity			
1	948 (40.7)^ab^	197 (62.7)^a^	2477 (46.9)
≥2	1379 (59.3)	117 (37.3)	2805 (53.1)
Parity			
1	2100 (90.2)^a^	292 (93.0)^a^	4328 (81.9)
≥2	227 (9.8)	22 (7.0)	954 (18.1)
Maternal education			
<Secondary school	71 (3.1)^a^	6 (1.9)	103 (1.9)
Secondary school	943 (40.5)^ab^	98 (31.2)^a^	1293 (24.5)
College graduate	1235 (53.1)^a^	187 (59.6)^a^	3560 (67.4)
Post-graduate	78 (3.4)^ab^	23 (7.3)	326 (6.2)
Caesarean Section	2117 (91.0)^ab^	209 (66.6)	3210 (60.8)
Previous cesarean delivery			
Yes	64 (2.8)^a^	10 (3.2)^a^	431 (8.2)
No	2263 (97.2)	304 (96.8)	4851 (91.8)
Pre-pregnant BMI	21.69 ± 2.82^a^	21.58 ± 2.73^a^	20.71 ± 2.74
Birth plurality			
Singletons	1450 (62.3)^a^	209 (66.6)^a^	5193 (98.3)
Twins	877 (37.7)	105 (33.4)	89 (1.7)

Note: Values are n (%) or mean (±standard deviation). ART, assisted reproductive technology; IVF, *in vitro* fertilization; ICSI, intracytoplasmic sperm injection; BMI, body mass index. Bonferroni corrected p-value = 0.016. ^a^Significantly different from control group (P < 0.016). ^b^Significantly different from ICSI group (P < 0.016).

**Table 2:** Incidence of Pregnancy and Perinatal Complications in ART and Spontaneously Pregnant Groups with Stratified Analysis by Birth Plurality.

	Total				Singletons				Twins			
	ART (n = 2641)	Controls (n = 5282)	P	Adjusted OR (95% CI)	ART (n = 1659)	Controls (n = 5193)	P	Adjusted OR (95% CI)	ART (n = 982)	Controls (n = 89)	P	Adjusted OR (95% CI)
**Pregnancy complications**												
GDM	309 (11.7)	342 (6.5)	<0.001	1.99 (1.69–2.36)	214 (12.9)	334 (6.4)	<0.001	2.23 (1.85–2.69)	95 (9.7)	8 (9.0)	1	—
Gestational hypertension	249 (9.4)	189 (3.6)	<0.001	2.58 (2.11–3.15)	124 (7.5)	183 (3.5)	<0.001	1.99 (1.56–2.53)	125 (12.7)	6 (6.7)	0.127	—
Preeclampsia	98 (3.7)	110 (2.1)	<0.001	1.49 (1.12–1.98)	43 (2.6)	107 (2.1)	0.21	1.71 (1.34–2.19)	55 (5.6)	3 (3.4)	0.471	—
mild preeclampsia	35 (1.3)	56 (1.1)	0.315	—	15 (0.9)	55 (1.1)	0.675	0.60 (0.33–1.09)	20 (2.0)	1 (1.1)	1	—
severe preeclampsia	63 (2.5)	54 (1.0)	<0.001	2.04 (1.40–2.96)	28 (1.7)	52 (1.0)	0.035	1.52 (0.95–2.43)	35 (3.6)	2 (2.3)	0.763	—
ICP	302 (11.4)	228 (4.3)	<0.001	2.86 (2.39–3.42)	110 (6.6)	214 (4.1)	<0.001	1.59 (1.25–2.02)	192 (19.6)	14 (15.7)	0.482	—
**Perinatal complications**												
Placenta previa	185 (7.0)	179 (3.4)	<0.001	2.23 (1.79–2.78)	117 (7.1)	175 (3.4)	<0.001	2.25 (1.75–2.89)	68 (6.9)	4 (4.5)	0.508	—
complete placenta previa	58 (2.2)	53 (1.0)	<0.001	2.61 (1.78–3.83)	44 (2.7)	53 (1.0)	<0.001	3.09 (2.05–4.66)	14 (1.4)	0	—	—
partial placenta previa	12 (0.5)	8 (0.2)	0.016	3.39 (1.36–8.46)	10 (0.6)	7 (0.1)	0.002	5.15 (1.92–13.82)	2 (0.2)	1 (1.1)	0.229	—
marginal placenta previa	115 (4.4)	118 (2.2)	<0.001	1.85 (1.42–2.41)	63 (3.8)	115 (2.2)	0.001	1.60 (1.17–2.19)	52 (5.3)	3 (3.4)	0.616	—
Placental abruption	40 (1.5)	16 (0.3)	<0.001	5.06 (2.83–9.06)	21 (1.3)	15 (0.3)	<0.001	4.43 (2.28–8.61)	19 (1.9)	1 (1.1)	1	—
pPROM	258 (9.8)	176 (3.3)	<0.001	3.05 (2.48–3.74)	85 (5.1)	161 (3.1)	<0.001	1.68 (1.27–2.22)	173 (17.6)	15 (16.6)	1	—
Abnormal placental cord insertion	105 (4.0)	167 (3.2)	0.067	1.37 (1.06–1.76)	69 (4.2)	163 (3.1)	0.051	1.40 (1.05–1.87)	36 (3.7)	4 (4.5)	0.568	—
Placental adherence	197 (7.5)	173 (3.3)	<0.001	2.37 (1.90–2.95)	115 (6.9)	168 (3.2)	<0.001	2.21 (1.71–2.84)	82 (8.4)	5 (5.6)	0.541	—
Postpartum haemorrhage	197 (7.5)	155 (2.9)	<0.001	2.72 (2.18–3.41)	82 (4.9)	151 (2.9)	<0.001	1.74 (1.30–2.32)	115 (11.7)	4 (4.5)	0.025	2.819 (1.02–7.83)
Polyhydramnios	60 (2.3)	73 (1.4)	0.005	1.79 (1.26–2.53)	36 (2.2)	71 (1.4)	0.03	1.65 (1.09–2.50)	24 (2.4)	2 (2.3)	1	—
Oligohydramnios	127 (4.8)	344 (6.5)	0.002	0.67 (0.54–0.83)	116 (7.0)	342 (6.6)	0.572	—	11 (1.1)	2 (2.3)	0.295	—

Note: CI, confidence interval; OR, odds ratio; Data are n (%). ART, assisted reproductive technology; GDM, gestational diabetes mellitus; ICP, intrahepatic cholestasis of pregnancy; pPROM, preterm premature rupture of membranes.

**Table 3:** Neonatal Outcomes in ART and Spontaneously Pregnant Groups with Stratified Analysis by Birth Plurality.

	Total			Singletons			Twins		
	ART (n = 2641)	Controls (n = 5282)	P	ART (n = 1659)	Controls (n = 5193)	P	ART (n = 982)	Controls (n = 89)	P
Preterm labour	1024/2641 (38.8)	556/5282 (10.5)	<0.001	291/1659 (17.5)	489/5193 (9.4)	<0.001	733/982 (74.6)	67/89 (75.3)	1
Low birthweight	1321/3623 (36.5)	430/5371 (8.0)	<0.001	170/1659 (10.2)	314/5193 (6.0)	<0.001	1151/1964 (58.6)	116/178 (65.2)	0.11
Macrosomia	121/3623 (3.3)	387/5371 (7.2)	<0.001	120/1659 (7.2)	387/5193 (7.5)	0.788	1/1964 (0.0)	0 (0.0)	—
Small for date infant	137/3623 (3.8)	63/5371 (1.2)	<0.001	17/1659 (1.0)	43/5193 (0.8)	0.451	120/1964 (6.1)	20/178 (11.2)	0.016
1 minute Apgar ≤ 7	178/3623 (4.9)	159/5371 (3.0)	<0.001	64/1659 (3.9)	152/5193 (2.9)	0.063	114/1964 (5.8)	7/178 (3.9)	0.675
5 minute Apgar ≤ 7	43/3623 (1.2)	13/5371 (0.2)	<0.001	11/1659 (0.7)	13/5193 (0.3)	0.028	32/1964 (1.6)	0 (0.0)	—

Note: Data are n (%).

**Table 4:** Incidence of Pregnancy and Perinatal Complications in IVF, ICSI and Spontaneously Pregnant Groups.

	ART (n = 2641)		Controls (n = 5282)
	IVF (n = 2327)	ICSI (n = 314)	
**Pregnancy complications**			
GDM	272 (11.7)^a^	37 (11.8)^a^	342 (6.5)
Gestational hypertension	217 (9.3)^a^	32 (10.2)^a^	189 (3.6)
Preeclampsia	92 (4.0)^a^	6 (1.9)	110 (2.1)
mild preeclampsia	32 (1.4)	3 (1.0)	56 (1.1)
severe preeclampsia	60 (2.6)^a^	3 (1.0)	54 (1.0)
ICP	274 (11.8)^a^	28 (8.9)^a^	228 (4.3)
**Perinatal complications**			
Placenta previa	166 (7.1)^a^	19 (6.1)	179 (3.4)
complete placenta previa	52 (2.2)^a^	6 (1.9)	53 (1.0)
partial placenta previa	11 (0.5)	1 (0.3)	8 (0.2)
marginal placenta previa	103 (4.4)^a^	12 (3.8)	118 (2.2)
Placental abruption	37 (1.6)^a^	3 (1.0)	16 (0.3)
pPROM	231 (9.9)^a^	27 (8.6)^a^	176 (3.3)
Abnormal placental cord insertion	90 (3.9)	15 (4.8)	167 (3.2)
Placental adherence	180 (7.7)^a^	17 (5.4)	173 (3.3)
Postpartum haemorrhage	171 (7.3)^a^	26 (8.3)^a^	155 (2.9)
Polyhydramnios	52 (2.2)^a^	8 (2.5)	73 (1.4)
Oligohydramnios	110 (4.7)^a^	17 (5.4)	344 (6.5)

Note: Data are n (%). IVF, *in vitro* fertilization; ICSI, intracytoplasmic sperm injection; GDM, gestational diabetes mellitus; ICP, intrahepatic cholestasis of pregnancy; pPROM, preterm premature rupture of membranes. Bonferroni corrected p-value = 0.016. ^a^Significantly different from control group (P < 0.016).

**Table 5:** Neonatal Outcomes in IVF, ICSI and Spontaneously Pregnant Groups.

	ART (n = 2641)		Controls (n = 5282)
	IVF (n = 2327)	ICSI (n = 314)	
Preterm labour	929/2327 (39.9)^ab^	95/314 (30.3)	556/5282 (10.5)
Low birthweight	1186/3204 (37.0)^a^	135/419 (32.2)^a^	430/5371 (8.0)
Macrosomia	106/3204 (3.3)^a^	15/419 (3.6)^a^	387/5371 (7.2)
Small for date infant	114/3204 (3.6)^a^	23/419 (5.5)^a^	63/5371 (1.2)
1 minute Apgar ≤ 7	163/3204 (5.1)^a^	15/419 (3.6)	159/5371 (3.0)
5 minute Apgar ≤ 7	29/3204 (0.9)^a^	3/419 (0.7)	13/5371 (0.2)

Note: Data are n (%). IVF, *in vitro* fertilization; ICSI, intracytoplasmic sperm injection. IVF group (3204 infants) consisted of 1450 cases of singleton gestation and 877 cases of twin gestation; ICSI group (419 infants) consisted of 209 cases of singleton gestation and 105 cases of twin gestation; Control group (5371 infants) consisted of 5193 cases of singleton gestation and 89 cases of twin gestation. Bonferroni corrected p-value = 0.016. ^a^Significantly different from control group (P < 0.016). ^b^Significantly different from ICSI group (P < 0.016).

**Table 6:** Incidence of Pregnancy and Perinatal Complications in Nulliparous and Multiparous Groups.

	Nulliparous Group			Multiparous Group		
	ART (n = 2392)	Controls (n = 4328)	P	ART (n = 249)	Controls (n = 954)	P
**Pregnancy complications**						
GDM	260 (10.9)	269 (6.2)	<0.001	49 (19.7)	73 (7.7)	<0.001
Gestational hypertension	219 (9.2)	142 (3.3)	<0.001	30 (12.0)	47 (4.9)	<0.001
Preeclampsia	94 (3.9)	78 (1.8)	<0.001	4 (1.6)	32 (3.4)	0.208
mild preeclampsia	35 (1.5)	43 (1.0)	0.096	0	13 (1.4)	—
severe preeclampsia	59 (2.5)	35 (0.8)	<0.001	4 (1.6)	19 (2.0)	1
ICP	281 (11.7)	181 (4.2)	<0.001	21 (8.4)	47 (4.9)	0.044
**Perinatal complications**						
Placenta previa	162 (6.8)	113 (2.6)	<0.001	23 (9.2)	65 (6.8)	0.218
complete placenta previa	47 (2.0)	26 (0.6)	<0.001	11 (4.4)	27 (2.8)	0.221
partial placenta previa	10 (0.4)	5 (0.1)	0.015	2 (0.8)	3 (0.3)	0.227
marginal placenta previa	105 (4.4)	82 (1.9)	<0.001	10 (4.0)	36 (3.8)	0.853
Placental abruption	36 (1.5)	11 (0.3)	<0.001	4 (1.6)	5 (0.5)	0.094
pPROM	236 (9.9)	114 (2.6)	<0.001	22 (8.8)	62 (6.5)	0.209
Abnormal placental cord insertion	95 (4.0)	132 (3.0)	0.048	10 (4.0)	35 (3.7)	0.851
Placental adherence	170 (7.1)	117 (2.7)	<0.001	27 (10.8)	56 (5.9)	0.011
Postpartum haemorrhage	171 (7.1)	97 (2.2)	<0.001	26 (10.4)	58 (6.1)	0.024
Polyhydramnios	51 (2.1)	50 (1.2)	0.002	9 (3.6)	23 (2.4)	0.275
Oligohydramnios	113 (4.7)	306 (7.1)	<0.001	14 (5.6)	38 (4.0)	0.292

Note: Data are n (%). ART, assisted reproductive technology; GDM, gestational diabetes mellitus; ICP, intrahepatic cholestasis of pregnancy; pPROM, preterm premature rupture of membranes.

**Table 7:** Neonatal Outcomes in Nulliparous and Multiparous Groups.

	Nulliparous Group			Multiparous Group		
	ART (n = 2392)	Controls (n = 4328)	P	ART (n = 249)	Controls (n = 954)	P
Preterm labour	919/2392 (39.4)	348/4328 (8.0)	<0.001	105/249 (42.2)	208/954 (21.8)	<0.001
Low birthweight	1210/3291 (36.8)	298/4401 (6.8)	<0.001	111/332 (33.4)	132/970 (13.6)	<0.001
Macrosomia	109/3291 (3.3)	312/4401 (7.1)	<0.001	12/332 (3.6)	75/970 (7.7)	0.01
Small for date infant	130/3291 (4.0)	55/4401 (1.2)	<0.001	7/332 (2.1)	8/970 (0.8)	0.073
1 minute Apgar ≤ 7	161/3291 (4.9)	112/4401 (2.5)	<0.001	17/332 (5.1)	47/970 (4.8)	0.883
5 minute Apgar ≤ 7	43/3291 (1.3)	6/4401 (0.1)	<0.001	0	7/970 (0.7)	—

Note: Data are n (%). Nulliparous Group of ART (3291 infants) group consisted of 1493 cases of singleton gestation and 899 cases of twin gestation; Nulliparous group of control group (4401 infants) consisted of 4255 cases of singleton gestation and 73 cases of twin gestation; Multiparous group of ART group (322 infants) consisted of 166 cases of singleton gestation and 83 cases of twin gestation; Multiparous group of control group (970 infants) consisted of 938 cases of singleton gestation and 16 cases of twin gestation.

**Table 8:** Incidence of Pregnancy, Perinatal Complications and Neonatal Outcomes in Singleton Spontaneously Conceived Pregnancies in Nulliparous Women to those who Conceived with ART.

	Singletons of ART Nulliparas (n = 1493)	Singletons of Control Nulliparas (n = 4255)	P
**Pregnancy complications**			
GDM	177 (11.9)	262 (6.2)	<0.001
Gestational hypertension	109 (7.3)	137 (3.2)	<0.001
Preeclampsia	43 (2.9)	76 (1.8)	0.015
mild preeclampsia	15 (1.0)	42 (1.0)	1
severe preeclampsia	28 (1.9)	34 (0.8)	0.001
ICP	100 (6.7)	169 (4.0)	<0.001
**Perinatal complications**			
Placenta previa	101 (6.8)	111 (2.6)	<0.001
complete placenta previa	36 (2.4)	26 (0.6)	<0.001
partial placenta previa	8 (0.5)	4 (0.1)	0.004
marginal placenta previa	57 (3.8)	81 (1.9)	<0.001
Placental abruption	19 (1.3)	11 (0.3)	<0.001
pPROM	75 (5.0)	103 (2.4)	<0.001
Abnormal placental cord insertion	62 (4.2)	130 (3.1)	0.045
Placental adherence	96 (6.4)	112 (2.6)	<0.001
Postpartum haemorrhage	62 (4.2)	94 (2.2)	<0.001
Polyhydramnios	30 (2.0)	49 (1.2)	0.019
Oligohydramnios	105 (7.0)	305 (7.2)	0.907
**Neonatal Outcomes**			
Preterm labour	255 (17.1)	294 (6.9)	<0.001
Low birthweight	154 (10.3)	204 (4.8)	<0.001
Macrosomia	108 (7.2)	312 (7.3)	0.954
Small for date infant	15 (1.0)	40 (0.9)	0.877
1 minute Apgar ≤ 7	56 (3.8)	106 (2.5)	0.014
5 minute Apgar ≤ 7	11 (0.7)	6 (0.1)	0.001

Note: Data are n (%). ART, assisted reproductive technology; GDM, gestational diabetes mellitus; ICP, intrahepatic cholestasis of pregnancy; pPROM, preterm premature rupture of membranes.
