# Supplementary material for: Maternal and Live-birth Outcomes of Pregnancies following Assisted Reproductive Technology: A Retrospective Cohort Study
Source: Sci Rep. 2016 Oct 20;6:35141. doi: 10.1038/srep35141 (PMC5071829; doi:10.1038/srep35141)
Supplement: Supplementary Information [file srep35141-s1.doc]

**Maternal and Live-birth Outcomes of Pregnancies following Assisted Reproductive Technology: A Retrospective Cohort Study.**

Linling Zhu^1,2#^, Yu Zhang^1,2#^, Yifeng Liu^1,2^, Runjv Zhang^1,2^, Yiqing Wu^1,2^, Yun Huang^1,2^, Feng Liu^1^, Meigen Li^1^, Saijun Sun^1^, Lanfeng Xing^1^, Yimin Zhu^1,2^, Yiyi Chen^1^, Li Xu^1^, Liangbi Zhou^1^, Hefeng Huang^1,2^*, Dan Zhang^1,2^*

^#^These authors contributed equally to this work.

1.Department of Reproductive Endocrinology, Women’s Hospital, School of Medicine, Zhejiang University, Hangzhou, Zhejiang, 310006, P. R. China

2. Key Laboratory of Reproductive Genetics (Ministry of Education), Zhejiang University, Hangzhou, 310006, P. R. China

*Corresponding author: Dan Zhang (E-mail: zhangdan@zju.edu.cn; Tel: 86-571-89992081), and Hefeng Huang (E-mail: huanghefg@hotmail.com)

**Supplementary Table S1. Clinical definition of the complications and outcomes**

| **Complications and outcomes** | **Clinical definitions** |
| --- | --- |
| **Pregnancy complications** |  |
| Gestational diabetes mellitus | Perform a diagnostic 75-g OGTT (oral glucose tolerance test) at 24th-28th week of gestation in pregnant women not previously diagnosed with diabetes mellitus, and the cut points of glucose values of 75-g OGTT: 0 h, 5.1mmol/L; 1 hour, 10.0 mmol/L; 2 hours, 8.5 mmol/L. The diagnosis of GDM can be made when any one value is met or exceeded[^1^](#_ENREF_1). |
| Gestational hypertension | New onset of hypertension (systolic blood pressure greater than or equal to 140 mmHg and/or diastolic blood pressure greater than or equal to 90 mm Hg) after 20 weeks of gestation without any maternal or fetal features of preeclampsia, followed by return of blood pressure to normal within 3 months postpartum[^2^](#_ENREF_2). |
| Preeclampsia | Diagnostic criteria include the development of hypertension, defined as a persistent systolic blood pressure of 140 mm Hg or higher, or a diastolic blood pressure of 90 mm Hg or higher after 20 weeks of gestation in a women with previously normal blood pressure[^2^](#_ENREF_2). |
| mild preeclampsia | Preeclampsia with the absence of severe manifestations often has been characterized as "mild". |
| severe preeclampsia | A diagnosis of severe preeclampsia can be made when accompanied by one or more of the following conditions: systolic blood pressure of 160 mm Hg or higher, or diastolic blood pressure of 110 mm Hg or higher on two occasions at least 4 hours apart while the patient is on bed rest; thrombocytopenia; impaired liver function as indicated by abnormally elevated blood concentrations of liver enzymes, severe persistent right upper quadrant or epigastric pain unresponsive to medication and accounted for by alternative diagnoses, or both; progressive renal insufficiency; pulmonary edema; cerebral or visual disturbances. |
| Intrahepatic cholestasis of pregnancy | The presence of unexplained palm and sole pruritus and abnormal liver function during pregnancy which disappear after delivery without primary lesions on the skin or hepatobilitary diseases[^3^](#_ENREF_3). |
| **Perinatal complications** |  |
| Placenta previa | Placenta previa exists when the placenta is inserted wholly or in part into the lower segment of the uterus. It is classified by ultrasound imaging according to what is relevant clinically: if the placenta lies over the internal cervical os, it is considered a major previa; if the leading edge of the placenta is in the lower uterine segment but not covering the cervical os, minor or partial previa exists^4^. |
| Placental abruption | Placenta abruption, defined as a premature separation of a normally implanted placenta. The diagnosis of placental abruption is clinical, while ultrasound scan and cardiotocography are tools with limited use^5^. |
| Preterm premature rupture of membranes | Membrane rupture before labor and before 37 weeks of gestation is referred to as preterm premature rupture of membranes[^6^](#_ENREF_5). |
| Abnormal placental cord insertion | The placental cord insertion site is abnormal if the site is located at the edge of the placental disk (marginal cord insertion) or if the umbilical vessels separate from each other and course between the amnion and chorion before reaching the placenta (velamentous cord insertion)[^7^](#_ENREF_6). |
| Placental adherence | A pathology characterized by abnormal and firm attachment of the placenta to the myometrium[^8^](#_ENREF_7). |
| Postpartum haemorrhage | An estimated blood loss in excess of 500 mL following a vaginal birth or a loss of greater than 1,000 mL following cesarean birth[^9^](#_ENREF_8). |
| Polyhydramnios | The amniotic fluid is more than 2000 mL during pregnancy, with a prevalence rate of 0.5%–1% and no difference in the appearance and characteristics of amniotic fluid compared with normal amniotic fluid[^10^](#_ENREF_9). |
| Oligohydramnios | Oligohydramnios is defined as Amniotic Fluid Index (AFI) of <5 cm at any gestational age (GA) or AFI <5th percentile according to GA[^11^](#_ENREF_10). |
| **Infant outcomes** |  |
| Preterm labour | Gestational age at delivery ≥ 28 weeks and <37 weeks[^12^](#_ENREF_11). |
| Low birthweight | A birth weight of a liveborn infant of less than 2,500 g (5 pounds 8 ounces) regardless of gestational age^13^. |
| Macrosomia | The term fetal macrosomia implies fetal growth beyond a specific weight, usually 4,000 g (8 lb, 13 oz) or 4,500 g (9 lb, 4 oz), regardless of the fetal gestational age[^14^](#_ENREF_12). |
| Small for date infant | Infants whose term was between 37 weeks and 42 weeks, with the birth weight less than 2500g[^15^](#_ENREF_13). |

**Supplementary Table S2. Final equation of logistic regression.**

| **X1: ART; X2: Gravidity; X3: Parity; X4: pre-pregnant BMI; X5: Maternal education; X6:** **Previous cesarean delivery** | | |
| --- | --- | --- |
| Total | GDM | logit(P)=-5.212+0.69X1+0.52X3+0.438X4+0.368X5 |
|  | Gestational hypertension | logit(P)=-4.977+0.947X1+0.288X3+0.65X4 |
|  | Preeclampsia | logit(P)=-3.442+0.399X1+0.481X4-0.516X5 |
|  | mild preeclampsia | logit(P)=-2.204-1.637X3+0.611X4-0.715X5+1.844X6 |
|  | severe preeclampsia | logit(P)=-4.118+0.71X1+0.346X4-0.426X5 |
|  | ICP | logit(P)=-3.099+1.052X1 |
|  | Placenta previa | logit(P)=-3.787+0.803X1+0.299X2+0.357X3-0.198X5+0.409X6 |
|  | complete placenta previa | logit(P)=-6.222+0.961X1+1.259X3 |
|  | partial placenta previa | logit(P)=-6.678+1.221X1+1.26X6 |
|  | marginal placenta previa | logit(P)=-3.62+0.616X1+0.449X2-0.322X5 |
|  | Placental abruption | logit(P)=-5.796+1.622X1 |
|  | pPROM | logit(P)=-2.746+1.114X1+0.341X3-0.385X5 |
|  | Abnormal placental cord insertion | logit(P)=-4.03+0.312X1+0.196X5+0.594X6 |
|  | Placental adherence | logit(P)=-4.226+0.862X1+0.577X2+0.319X3-0.178X5 |
|  | Postpartum haemorrhage | logit(P)=-3.422+1.002X1+0.63X3-0.32X5 |
|  | Polyhydramnios | logit(P)=-5.108+0.58X1+0.679X3 |
|  | Oligohydramnios | logit(P)=-1.545-0.4X1-0.534X3-0.181X5 |
| Singletons | GDM | logit(P)=-5.247+0.802X1+0.522X3+0.439X4+0.377X5 |
|  | Gestational hypertension | logit(P)=-4.797+0.688X1+0.7X4+0.424X6 |
|  | Preeclampsia | logit(P)=-3.362+0.538X4-0.598X5 |
|  | mild preeclampsia | logit(P)=-2.557-0.513X1-1.517X3+0.805X4-0.787X5+1.755X6 |
|  | severe preeclampsia | logit(P)=-3.14+0.421X1-0.541X5 |
|  | ICP | logit(P)=-2.626+0.466X1-0.19X5 |
|  | Placenta previa | logit(P)=-3.681+0.809X1+0.281X2+0.384X3-0.243X5+0.417X6 |
|  | complete placenta previa | logit(P)=-6.214+1.128X1+1.266X3 |
|  | partial placenta previa | logit(P)=-6.833+1.638X1+1.41X6 |
|  | marginal placenta previa | logit(P)=-3.457+0.47X1+0.426X2-0.374X5 |
|  | Placental abruption | logit(P)=-5.844+1.487X1 |
|  | pPROM | logit(P)=-3.099+0.518X1+0.587X3-0.4X5 |
|  | Abnormal placental cord insertion | logit(P)=-3.126+0.337X1-0.239X2+0.582X6 |
|  | Placental adherence | logit(P)=-4.299+0.792X1+0.622X2+0.386X3-0.218X5 |
|  | Postpartum haemorrhage | logit(P)=-4.068+0.553X1+0.861X3+0.213X4-0.359X5 |
|  | Polyhydramnios | logit(P)=-5.429+0.502X1-0.498X2+0.941X3+0.371X4 |
|  | Oligohydramnios | logit(P)=-1.492-0.61X3-0.166X5 |
| Twins | Postpartum haemorrhage | logit(P)=-4.093+1.036X1 |

**References**

1. Practice Bulletin No. 137: Gestational diabetes mellitus. *Obstet Gynecol* **122**, 406-416 (2013).

2. Hypertension in pregnancy. Report of the American College of Obstetricians and Gynecologists' Task Force on Hypertension in Pregnancy. *Obstet Gynecol* **122**, 1122-1131 (2013).

3. [Guidelines for diagnosis and treatment of intrahepatic cholestasis of pregnancy (2015)]. *Zhonghua Fu Chan Ke Za Zhi* **50**, 481-485 (2015).

4. RCOG Release: Green-Top Guideline Published On Placenta Praevia, Placenta Praevia Accreta And Vasa Praevia, 2011.

5. Saccone, G., Maruotti, G.M., Paternoster, M. & Martinelli, P. Diagnosis of placental abruption: a legal issue for physicians. *J Matern Fetal Neonatal Med*, 1-2 (2016).

6. Practice Bulletin No. 160: Premature Rupture of Membranes. *Obstet Gynecol* **127**, e39-51 (2016).

7. Robinson, L.K., Jones, K.L. & Benirschke, K. The nature of structural defects associated with velamentous and marginal insertion of the umbilical cord. *Am J Obstet Gynecol* **146**, 191-193 (1983).

8. Miller, D.A., Chollet, J.A. & Goodwin, T.M. Clinical risk factors for placenta previa-placenta accreta. *Am J Obstet Gynecol* **177**, 210-214 (1997).

9. ACOG Practice Bulletin: Clinical Management Guidelines for Obstetrician-Gynecologists Number 76, October 2006: postpartum hemorrhage. *Obstet Gynecol* **108**, 1039-1047 (2006).

10. Volante, E., Gramellini, D., Moretti, S., Kaihura, C. & Bevilacqua, G. Alteration of the amniotic fluid and neonatal outcome. *Acta Biomed* **75 Suppl 1**, 71-75 (2004).

11. Leibovitch, L. et al. Short-term outcome among term singleton infants with intrapartum oligohydramnios. *Acta Paediatr* **101**, 727-730 (2012).

12. NICE. Preterm labour and birth. London: National Institute for Health and Care Excellence, 2015.

13. P07 - Disorders related to short gestation and low birth weightin ICD-10.

14. Chatfield, J. ACOG issues guidelines on fetal macrosomia. American College of Obstetricians and Gynecologists. *Am Fam Physician* **64**, 169-170 (2001).

15. Sam Bill, J.R. et al. [Incidence and influencing factors of term small for gestational age infants in China]. *Zhonghua Yi Xue Za Zhi* **96**, 48-52 (2016).
